# Elucidating Cancer Subtypes by Using the Relationship between DNA Methylation and Gene Expression

**DOI:** 10.3390/genes15050631

**Published:** 2024-05-16

**Authors:** Muneeba Jilani, David Degras, Nurit Haspel

**Affiliations:** 1 Department of Computer Science, University of Massachusetts Boston, Boston, MA 02125, USA; muneeba.jilani001@umb.edu; 2 Department of Mathematics, University of Massachusetts Boston, Boston, MA 02125, USA

**Keywords:** data integration, cancer subtypes, multi-omics

## Abstract

Advancements in the field of next generation sequencing (NGS) have generated vast amounts of data for the same set of subjects. The challenge that arises is how to combine and reconcile results from different omics studies, such as epigenome and transcriptome, to improve the classification of disease subtypes. In this study, we introduce sCClust (sparse canonical correlation analysis with clustering), a technique to combine high-dimensional omics data using sparse canonical correlation analysis (sCCA), such that the correlation between datasets is maximized. This stage is followed by clustering the integrated data in a lower-dimensional space. We apply sCClust to gene expression and DNA methylation data for three cancer genomics datasets from the Cancer Genome Atlas (TCGA) to distinguish between underlying subtypes. We evaluate the identified subtypes using Kaplan–Meier plots and hazard ratio analysis on the three types of cancer—GBM (glioblastoma multiform), lung cancer and colon cancer. Comparison with subtypes identified by both single- and multi-omics studies implies improved clinical association. We also perform pathway over-representation analysis in order to identify up-regulated and down-regulated genes as tentative drug targets. The main goal of the paper is twofold: the integration of epigenomic and transcriptomic datasets followed by elucidating subtypes in the latent space. The significance of this study lies in the enhanced categorization of cancer data, which is crucial to precision medicine.

## 1. Introduction

The cells in an organism have the same genome, but their function may be very different. The identity of a cell is determined by transcription regulators that are accountable for controlling the expression of genes. There are various regulators in this complex setup, including cis-regulatory elements and transcription factors (TFs). One of the major epigenetic mechanisms that control expression of genes is DNA methylation [1]. DNA methylation appears to control gene expression by a complex mechanism that involves various proteins and TFs [2,3]. Abundance of methylation has been associated with many disease phenotypes, including cancer [4]. DNA methylation is a constant process that controls tumorigenesis and regulates expression of genes in cancer cells; however, this mechanism needs more research [5]. Therefore, understanding the association between DNA methylation and gene expression datasets can be meaningful.

In precision oncology, subtyping of patients into groups is desired to identify effective treatment strategies according to well-separated molecular subgroups [6,7]. The approaches to subtyping can be classified into two broad categories: clustering using single-omics data and clustering using multi-omics data [8]. Within the multi-omics subtyping methods, depending on the stage at which the data are clustered, there are three types of approach [9]: The simplest approach, early clustering, combines all omics profiles followed by clustering [10]. An example of such an approach is the integrative probabilistic model by Wu et al. [10], which finds a shared principal subspace of different omics data types in order to cluster these data into cancer subtypes. Early clustering results in increased dimensionality and also disregards varying distribution of values in different types of omics data. The second approach, late clustering, groups each omics profile individually and then integrates the results. An example of such an approach is the perturbation clustering approach by Nguyen et al. [11], which uses connectivity matrices to co-cluster the subjects within a certain type of omics data, followed by integration of the matrices. This late-integration approach to subtyping ignores weak but consistent interactions among different types of omics data. The third category of multi-omics clustering consists of methods that either perform joint dimensionality reduction of omics profiles or similarity-based methods. These methods construct a single model to account for all types of omics data. The benchmark multi-omics methods used in our study for comparison lie in this category, and so does our proposed approach, sCClust.

Implicit genetic mechanisms can be uncovered by exploring the relationship between DNA methylation and gene expression datasets, but this task is not as simple as pairwise association. One of the reasons is the high dimensionality of these genomic data [12], i.e., the number of variables being much larger than the number of subjects. Due to the challenges in the analysis of high-dimensional data, many techniques have been proposed that attempt to counter the problem. Principal component analysis (PCA) [13] is one such method that attempts to lower the dimension of the data before further analysis attempts are made. However, due to the functional relationship between genes being non-linear, PCA cannot guarantee extraction of clinically and statistically relevant patterns form genetic data due to its linearity assumption [14]. A recent method by Zheng et al. [15] attempted to detect aberrant DNA methylation patterns in subjects and to further couple these patterns with gene expression alterations. This was a promising effort; however, statistically aggregating these datasets in a lower-dimensional space can result in revelation of inherent information and potentially aid improved subtyping [16]. In recent years, machine learning methods, such as convolutional neural networks, have utilized multi-omics for low-dimensional embedding and tumor staging [17,18].

Another issue to be highlighted is that due to the heterogeneity in gene regulation and the majority of analysis efforts targeting transcriptomic data, there is a need for methods that integrate DNA methylation and gene expression profiles from the same set of patients, in order to perform cancer classification. But, as mentioned above, this task is not trivial. Studying the relation between two variables in high-dimensional datasets one-at-a-time is biologically and statistically uninformative. Rather, if two variables are projected into a lower-dimensional space and then the relationship between the composite variables is investigated, it may reveal some interesting and novel insights into the data. Aggregating omics datasets in the form of low-dimensional composite variables can aid in overcoming the dimensionality curse in an effective manner as well as aid in making use of the relationship between various types of omics profiles.

**Figure 1:**
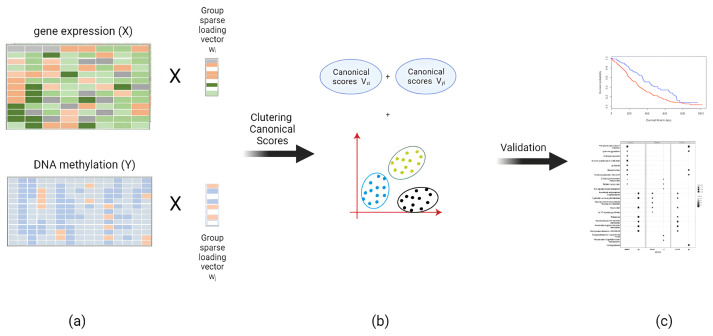
An overview of the methodology: (**a**) Sparse canonical correlation analysis is performed, in order to project the data to obtain canonical scores. (**b**) K-means clustering is performed. (**c**) validation is carried out, using Kaplan–Meier plots, followed by pathway analysis in the end.

In this study, we propose the use of sparse canonical correlation analysis [19], in order to combine DNA methylation and gene expression datasets while mapping them to a lower-dimensional space. This is followed by clustering the results of the integrated data, followed by clustering evaluation using Kaplan–Meier plots (Figure 1). The key goal is to get more separation and less overlap between the Kaplan–Meier plots compared to pre-existing single- and multi-omics studies, thus paving the way for effective personalized medicine for the treatment of many cancer types. We support our claims with hazard ratio analysis and the SEP criterion, which measures the average difference between hazard rates. We also perform pathway over-representation analysis to highlight potential drug targets for each identified subtype, followed by a short analysis of the canonical variables for gene expression datasets.

The rest of the paper is organized as follows: Section 2 comprises an overview of the studies that have attempted to perform high-dimensional data integration in genomics or related fields. This is followed by detailed description of the methods employed in Section 3. In Section 4, we describe and discuss the results of applying our method to three cancer genomics datasets from the TCGA. We summarize and conclude our findings at the end.

## 2. Materials and Methods

### 2.1. Data Preparation

We used mRNA and methylation datasets for glioblastoma multiforme (GBM), lung squamous cell carcinoma (LSCC) and colon adenocarcinoma (COAD), available on the TCGA website. Data containing DNA methylation and gene expression levels with a missing rate higher than 5% in any cancer type were discarded. The datasets were consolidated and subjects with both expression and methylation data available were retained. R was the language of choice for the data preprocessing [20].

For each type of cancer in this study, DNA methylation data comprised methylated sites for each subject in the dataset, whereas gene expression data consisted of gene expression levels for each subject in the dataset. A brief summary of the datasets is provided in the Supplementary Materials (Supplementary Table S1). The number of variables was much larger than the number of samples. For example, for the lung cancer dataset, the gene expression dimensionality is 12,042 and the DNA methylation dimensionality is 23,074 for 106 subjects; thus, dimensionality reduction was necessary for analysis and aggregation. Using the original datasets of gene expression and DNA methylation, a standard clustering algorithm is not an ideal choice for clustering, as the results will not be accurate, owing to the high dimensionality [21].

### 2.2. sCClust: Sparse Canonical Correlation Analysis with Clustering

**Figure 2:**
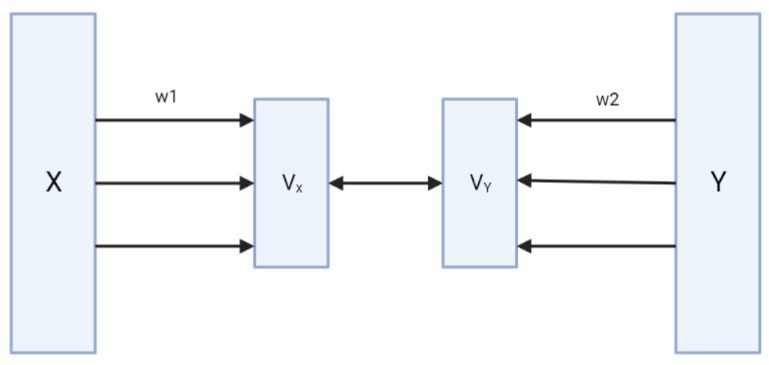
A simplistic depiction of the sparse canonical correlation analysis. The original variables *X* and *Y* are projected to $V_{x}$ and $V_{y}$ in the lower-dimensional space; $w_{1}$ and $w_{2}$ are canonical vectors; sCCA uses canonical variates with sparse coefficients to mirror the transcriptional regulatory mechanism.

In this study, we applied sparse canonical correlation analysis (sCCA) to the three TCGA datasets. We used the sparse version as canonical correlation analysis (CCA) [22] alone cannot be used on expression and methylation data without modification, due to the large data dimensionality. Since there are more variables than subjects in each dataset, infinitely linear combinations of variables in each dataset are perfectly correlated across the datasets. The solution here was regularization of the problem by imposing a penalty on the $l_{1}$ norm (sum of absolute values) of the linear combinations or canonical vectors. The sCCA variant of CCA (Figure 2) solves the problem of multicollinearity by using sparse loadings in the CCA algorithm [23,24]. The sCCA optimization problem is as follows:

$$\max_{w_{1},w_{2}}Cov\left(Xw_{1},Yw_{2}\right)-\tau_{1}∥w_{1}{∥}_{1}-\tau_{2}{∥w_{2}∥}_{1}$$

subject to the constraints $Var(Xw_{1})=1$ and $Var(Yw_{2})=1$, where *X* and *Y* are the data matrices (cases in rows, variables in columns), $w_{1}$ and $w_{2}$ are the weights of the linear combinations that define the canonical variables $Xw_{1}$ and $Yw_{2}$, ${∥\cdot ∥}_{1}$ is the $l_{1}$ norm, and $\tau_{1},\tau_{2}$ are positive regularization parameters that specify the trade-off between the fit to the data and the sparsity of the vectors $w_{1}$ and $w_{2}$. The maximization of the covariance $Cov(Xw_{1},Yw_{2})$ under the variance constraints $Var\left(Xw_{1}\right)=Var\left(Yw_{2}\right)=1$ is equivalent to maximizing the correlation $Cor(Xw_{1},Yw_{2})$ while also enabling the inclusion of $l_{1}$ penalty terms. The solution of the sCCA optimization problem is found through iterative methods [25].

From a biology standpoint, sCCA is more in line with transcriptional machinery. Since a small percentage of genes is involved under a specific set of conditions, this is portrayed by the use of canonical variates with sparse coefficients in sCCA.

Various sCCA implementations have been proposed. Among the more popular ones are those of Waaijenborg et al. [24] and Witten et al. [23], which maximize the covariance. However, a limitation of covariance-maximizing methods is that two canonical variables that are only mildly correlated but have high variance can be selected over two canonical variables that are highly correlated but have low variance. Charlotte et al. [14] argued that in high-dimensional scenarios the methods that maximize correlation rather than covariance yield optimal results as they show more correlation and they focus less on variance in individual datasets. We analyzed the data using the aforementioned sCCA methods alongside the sCCA implementation by Csala et al. [19], which maximizes the correlation and uses the elastic net (ENet) regression model to resolve the multicollinearity problem. The latter approach improves the interpretability of the results by setting some weights to zero [26]. With the modification outlined below, this implementation of sCCA yielded better results than the others. Hence, we retained it for our analysis.

The R package *sRDA* was used to carry out sCCA in our analysis. We modified the main sCCA function so that it would accept two sparsity arguments instead of one. The rationale behind this alteration is that using a common sparsity parameter for both datasets results in retaining the same number of variables for each dataset to form the canonical components. This is not desirable because the sparsity levels of canonical variables are in general different across datasets. Thus, we tweaked the function to allow separate penalty parameters for each dataset.

After jointly reducing the dimension of the DNA methylation and gene expression data, we identified cancer subtypes by clustering the reduced data with the k-means algorithm [27]. We use the abbreviation sCClust to designate our proposed approach based on sCCA and clustering.

### 2.3. Kaplan–Meier Plots and Minimum Hazard Ratio

After applying sCClust to the data, we performed survival analysis to evaluate the results. Survival is the percentage of entities or units that survive from the original data over time. The Kaplan–Meier (KM) plot [28,29] is a graphical display of survival data that represents the proportion of patients surviving against time as a step function. Steps signify occurrence of an event: each time there is a death, we see a dip in the KM plot.

A common measure of the separation between KM plots is the minimum hazard ratio $HR_{min}$ [30,31]. This is a measure of the differences between groups (here, cancer subtypes) in Cox’s proportional hazards model. Hazard ratios are typically used to measure the magnitude of separation between survival curves. The hazard rate [32,33] is the rate of death of an item of a given age, and it is denoted by λ. The hazard ratio is the ratio of hazard rates, i.e., if $\lambda_{1}$ is the hazard of the first subtype $l_{1}$ and if $\lambda_{2}$ is the hazard of the second subtype $l_{2}$ then the hazard ratio is

$$\lambda \left(l_{1},l_{2}\right)=\lambda_{1}\left(t\right)/\lambda_{2}\left(t\right)$$

When the number *n* of subtypes is greater than two, the minimum hazard rate is defined as

$$HR_{min}=\min\left\{\max\left(\lambda \left(l_{i},l_{j}\right),\lambda \left(l_{j},l_{i}\right)\right):1\leq i<j\leq n\right\}$$

In order to incorporate hazard ratios in a survival analysis, these ratios must stay constant over time, i.e., the assumption of proportional hazards must hold [34]. To verify this, we applied the global Schoenfeld test (GST) [35], a hypothesis test based on the correlation between Schoenfeld residuals and ranked event times. Small correlation statistics, with a probability larger than the significance level, lead to retaining the proportional hazard assumption [36].

In addition to the minimum hazard ratio, we employed the SEP statistic of Royston et al. [37] to measure the average difference between hazard rates. Denoting by ${\hat{\beta }}_{i}$ the coefficient estimate for the *i*-th subtype in Cox’s proportional hazards model and by $n_{i}$ the associated number of patient samples, such that $\sum_{i=1}^{C}n_{i}{\hat{\beta }}_{i}=0$ and $\sum_{i=1}^{C}n_{i}=n$, the SEP statistic is defined as $\exp(-\sum_{i=1}^{C}\left(n_{i}/n\right)|{\hat{\beta }}_{i}|)$.

## 3. Results

### 3.1. Survival Analysis

Using sCClust, sCCA was applied to the gene expression and DNA methylation measurements of each cancer dataset to obtain a lower-dimensional representation of these data. For instance, in the case of GBM, the original dataset containing 12,042 gene expression levels and 13,050 methylated sites for 211 subjects was reduced to a vector containing 211 canonical scores in the lower-dimensional space, thus facilitating clustering. Different combinations of sparsity parameters and numbers of canonical components were systematically assessed by grid search to maximize $HR_{min}$. We then combined the resulting low-dimensional canonical scores and clustered them with the *k*-means algorithm [27].

We estimated the optimal number of clusters by visual inspection of elbow plots [38] for aggregated and scaled scores (Supplementary Figure S1). Our choice of number of clusters was also guided by silhouette scores [39] and the ratio of the sum of the squares between clusters by the sum of the squares within clusters, which is a common statistical criterion for cluster evaluation [40]. We obtained three clusters for GBM and COAD each, and four for LSCC. These numbers of clusters aligned with the well-established subtypes of their respective cancer dataset [41,42,43]. The cluster sizes for each cancer dataset are provided in the Supplementary Materials (Supplementary Table S2).

**Figure 3:**
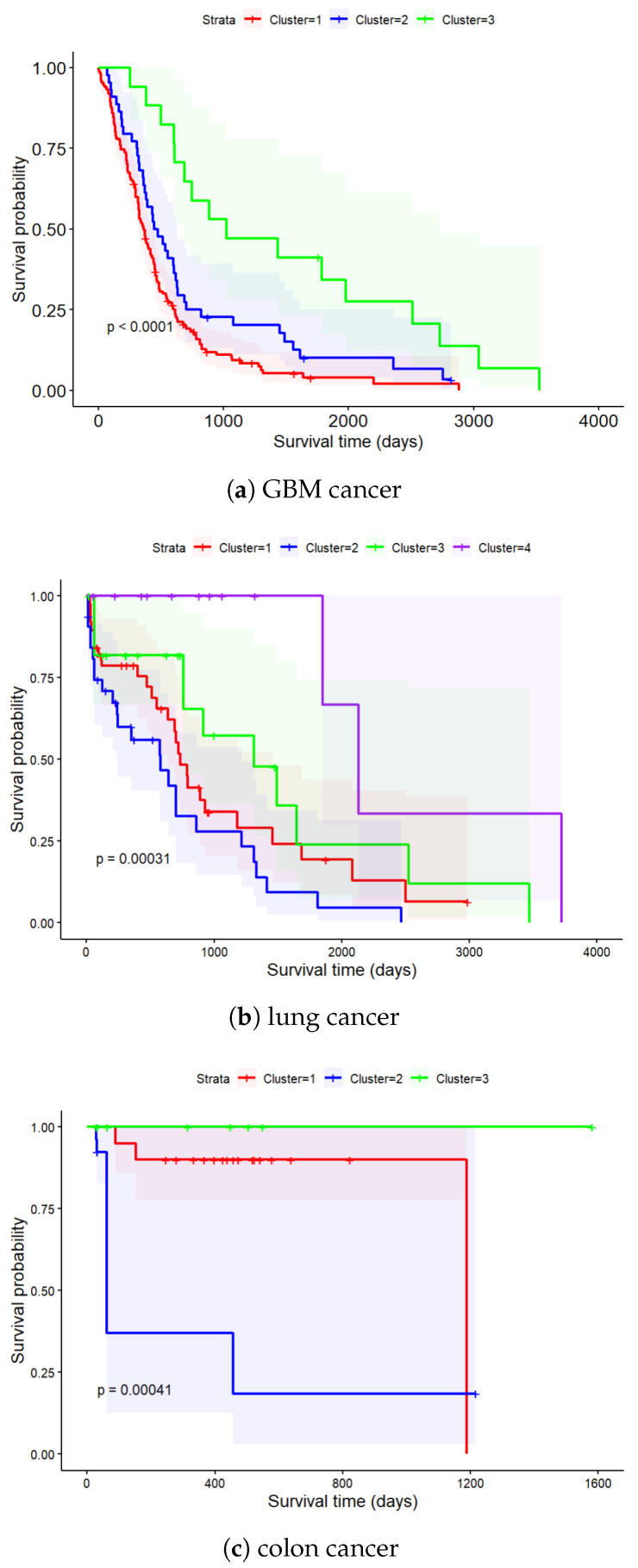
Survival probability as a function of time for different clusters/subtypes in GBM, LSCC and COAD cancer. The corresponding log-rank survival *p*-values are stated and the confidence interval is indicated by the shaded region.

Following the cluster analysis, KM plots were made for every cluster obtained from the corresponding cancer datasets. The efficacy for each subtype was measured by the separation within the corresponding KM curves. Furthermore, clustering and survival analysis was performed after the data were integrated in the lower-dimensional space. A comparison of the integrated clustering and the single-omics clustering approaches is presented below (Section 3.2). Figure 3 shows the KM curves for GBM, lung and colon cancers, respectively. The various colors illustrate different subtypes of cancer. The greater the distance between KM curves, the better the subtyping [44]. All identified subtypes were statistically significant with log-rank *p*-values ≤0.05.

The $HR_{min}$ and SEP criteria for cluster separation are shown in Table 1. For the GBM, LSCC and COAD datasets, we obtained $HR_{min}$ values of 1.4907, 1.6106 and 8.7327 and SEP values of 1.2889, 1.7026 and 1041.07, respectively, thus indicating greater separation between the KM plots compared to the other methods (see below). In all the model fits we obtained GST *p*-values >0.05, thus making the analysis valid for all the statistically significant subtypes (*p*-value ≤0.05).

### 3.2. Comparison with Single- and Multi-Omics Methods

The results of the analysis were compared with two classes of representative multi-omics and single-omics subtyping approaches. Similarity network fusion (SNF) [45] is a state-of-the-art method that builds similarity networks for all data sources and integrates them non-linearly. The rationale behind selecting SNF for comparison with our technique was its popularity and widely demonstrated usefulness, as well as the public availability of the code [46]. Another reason was the ability of SNF to combine data beyond visual integration, which is more in line with our methodology as compared to genomic browsers [47,48]. We implemented SNF using the R package *CancerSubtypes* [49]. Table 1 provides a side-by-side comparison of the proposed methodology with SNF for combining gene expression and DNA methylation data. We report hazard ratios as well as log-rank *p*-values. The number of clusters was kept the same as well-established subtypes of each cancer, three for GBM and colon cancer, and four for lung cancer [41,42,43]. Systematic testing was performed with different values of the tuning parameter α, which denotes the exponential similarity kernel, and the results with the largest values of $HR_{min}$ were retained. Aggregation using sCCA demonstrated better performance in terms of separation in KM plots with lower *p*-values. The method also resulted in larger $HR_{min}$ values for all three datasets analyzed in this study, as well as better performance in terms of SEP measure. In order to consider more recent multi-omics methods we compared sCClust with NEMO [9] and PIntMF [50]. NEMO (neighborhood-based multi-omics clustering) uses prior similarity-based multi-omics methods to build an inter-patient similarity matrix for clustering. The PIntMF (penalized integrative matrix factorization) approach uses a matrix factorization model with sparsity to perform clustering. As indicated in the Table 1, sCClust performed better in terms of different hazard measures when compared with these two multi-omics methods.

In single-omics cancer molecular subtyping, the majority of the efforts are focused on gene expression data [51]. We compared the performance of the proposed method with a robust sparse implementation of the OTRIMLE clustering algorithm [52] that employs multivariate Gaussian distribution to cluster the data, using the R package *otrimle*. Genes with low variance were filtered out using the R package *GWENA*. This method requires the number of clusters to be passed as input. We kept the same number of clusters as for the proposed and SNF approaches. Again, we performed systematic testing using a grid of tuning parameters, γ and *m*, which denote eigenratio constraint and the number of projections, respectively. The results yielding the largest value of $HR_{min}$ were selected. As shown in Table 1, our methodology produced lower log-rank *p*-values and greater $HR_{min}$ and SEP values, indicating more statistically significant differences and larger separation between subtypes. This illustrates the ability of the proposed method to yield better clinical association in comparison with single-omics and multi-omics classification of the subtypes.

### 3.3. Pathway Over-Representation Analysis

Pathway over-representation analysis is a technique used to identify biological processes that are enriched in an experimentally obtained list of genes [53]. In cancer analysis, these identified hallmarks of tumor traits can aid in better understanding of the drug susceptibility of the disease. Core genes in pathways can lead to disease phenotype discrimination [54].

Pathway over-representation analysis was performed after the identification of subtypes for the purpose of elucidating biological attributes associated with them. The analysis resulted in the identification of up-regulated and down-regulated genes with the help of the R packages *limma* and *clusterProfiler*.

**Figure 4:**
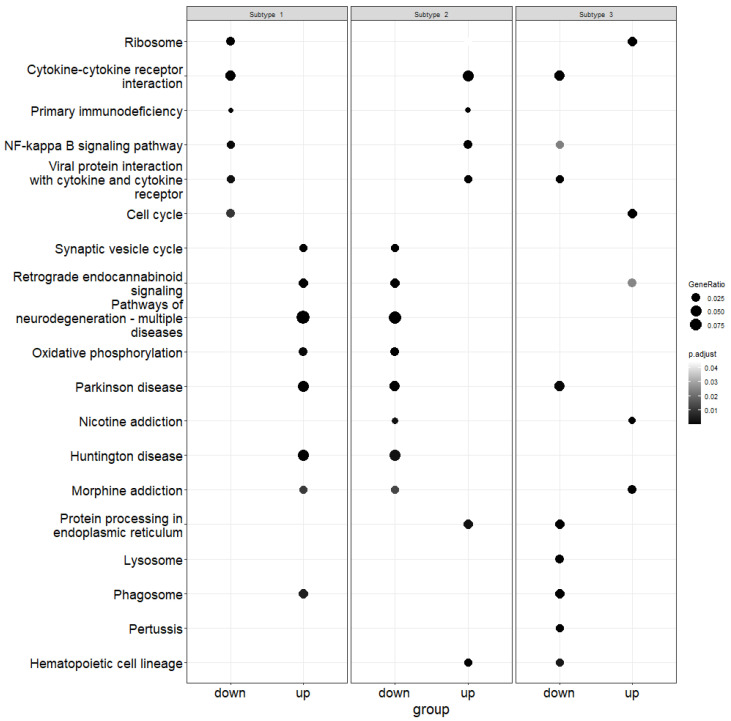
Illustration of pathway over-representation analysis for each subtype in the GBM dataset. The size of each point represents the proportion of genes in that pathway, whereas the gray shade indicates the *p*-value. The down column contains down-regulated genes and the up panel is for up-regulated genes.

**Figure 5:**
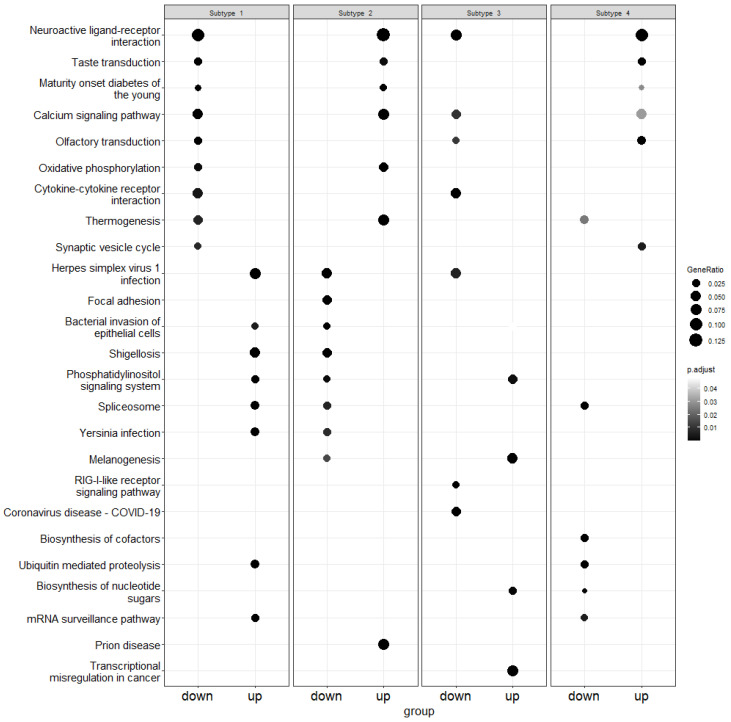
Illustration of pathway over-representation analysis for each subtype in the lung dataset. The size of each point represents the proportion of genes in that pathway whereas the gray shade indicates the *p*-value. The down column contains down-regulated genes and the up panel is for up-regulated genes.

**Figure 6:**
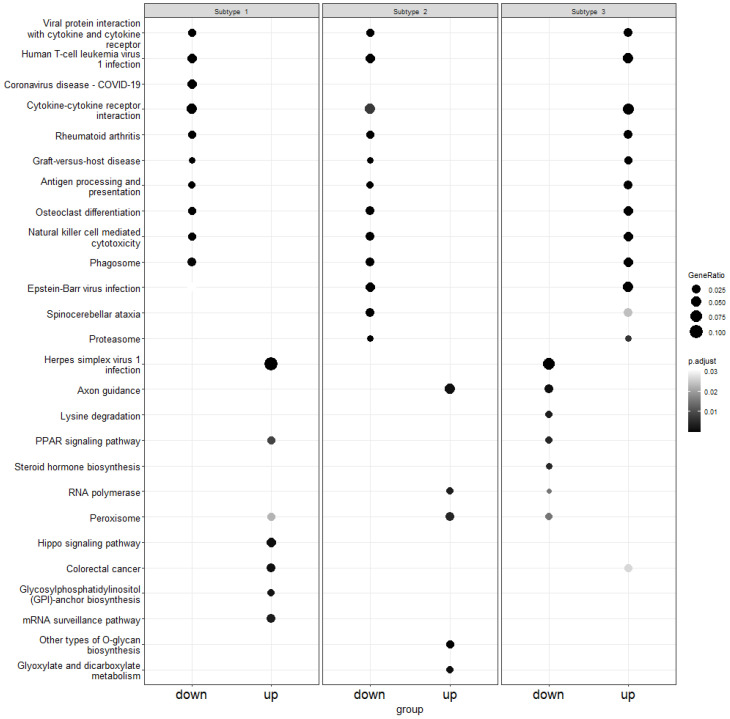
Illustration of pathway over-representation analysis for each subtype in the colon dataset. The size of each point represents the proportion of genes in that pathway whereas the gray shade indicates the *p*-value. The down column contains down-regulated genes and the up panel is for up-regulated genes.

The gene names were obtained for each subtype by mapping the canonical vectors back to the original expression data. Using enrichment analysis, we obtained the up and down-regulated genes. After that, pathway over-representation analysis was performed using the Kyoto Encyclopedia of Genes and Genomes (KEGG) database [55] in order to obtain pathways related to up and down-regulated genes. Figure 4, Figure 5 and Figure 6 demonstrate the results of this analysis on the GBM, lung and colon datasets, respectively.

For GBM, we identified 326 over-represented pathways. For subtype 1, 58 pathways were from up-regulated genes while 28 pathways were from down-regulated genes. Similarly, for subtype 2 and 3, 41 and 56 pathways were from up-regulated genes and 60 and 83 pathways were from down-regulated genes, respectively. As we can see in Figure 4, the cytokine–cytokine receptor interaction pathway was over-represented in all three subtypes. This pathway was reported to be an over-represented pathway in GBM [56], and genes in this pathway were demonstrated to be enriched in GBM [57]. The Parkinson’s disease pathway, which was also over-represented in all three subtypes, has been shown to have significant association with brain and thyroid cancer [58]. Similarly, the neurodegeneration pathway, which was over-represented due to up-regulated genes in the first subtype and due to down-regulated genes in the second subtype, was shown to be associated with GBM [59]. These figures highlight the disparity between the subtypes in terms of pathways that are over-represented.

For lung cancer, 372 over-represented pathways were identified. For each of the four subtypes identified, 70, 39, 29 and 26 pathways were from up-regulated genes and 51, 83, 35 and 39 pathways were from down-regulated genes, respectively. As illustrated (Figure 5), the neuroactive ligand–receptor interaction pathway was over-represented in all four subtypes. Genes significantly contributing to neuroactive ligand–receptor interactions pathway are highly relevant to lung cancer [60]. Another over-represented pathway, ubiquitin-mediated proteolysis, was due to up-regulated genes in the first subtype while it was due to down-regulated genes in the third subtype. This pathway is used to identify novel biomarkers and therapeutic targets in lung cancer [61]. Similarly, a calcium signaling pathway, which was over-represented in all the subtypes, contributes to lung cancer progression [62]. We can see that the subtypes are contrasting in terms of the altered pathways.

For colon cancer, we identified 312 over-represented pathways. For each of the three subtypes, 21, 16 and 94 pathways were from up-regulated genes and 87, 83 and 18 pathways were from down-regulated genes. As illustrated in Figure 6, the herpes simplex virus type 1 (HSV1) pathway was from down-regulated genes in the first subtype and from up-regulated genes in the third subtype. HSV1 is common in patients with colon cancer [63,64]. Similarly, the cytokine–cytokine receptor interaction pathway was over-represented in all three subtypes. The genes in this pathway were shown to be enriched for colon cancer [65]. The Epstein—Barr virus infection pathway, which was over-represented for subtype 2 and 3, is associated with the progression of colon cancer [66]. Again, we can see that not many over-represented pathways identified were shared between subtypes.

The main takeaway is that with such an analysis for each subtype, we can identify over-represented pathways alongside up and down-regulated genes. This could aid in drug treatment for the respective subtype. Pathway over-representation analysis also indicates that the subtypes identified vary when it comes to underlying over-represented pathways with corresponding up- and down-regulated genes.

### 3.4. Interpretation of the Canonical Weights of Genes

The examination of canonical weights for gene expression data indicates that sCCA selects biologically meaningful variables for their respective cancer datasets. The full list of genes selected by sCCA for each dataset is given in the Supplementary Materials (Supplementary Table S3). Below are some of the genes that were selected and their respective function:

*HOXA6* and *HOXA5* (homeobox A6 and A5) genes were selected by sCCA with top absolute average weights for GBM. Homeobox (HOX) genes play an important role in tissue homeostasis. Mutations in HOX genes lead to increased glioma predisposition [67,68]. *SOX10* (SRY-box transcription factor 10) is overly expressed in glioma [69]. It was selected with the third-highest absolute average weight. *HOXA7* (homeobox A7) was also selected by sCCA and *HOXA7* knockdown inhibits glioma cell migration [70].

For the lung cancer dataset, *PCGF1* (polycomb group ring finger 1) was selected with the highest absolute average weight. *PCGF1* is known to be a prognostic biomarker for many cancers, including lung cancer [71]. Next on the list was *BANF1* (barrier to auto-integration factor 1), which is a candidate marker of lung cancer patient prognosis [72]. This was followed by *FGF22* (fibroblast growth factor 22), which is also a prognostic biomarker for lung cancer [73]. *PRKCSH* (protein kinase C substrate 80K-H) was also selected by sCCA for the lung dataset, and this gene was shown to be significantly up-regulated in lung cancer tissues [74].

For the colon cancer dataset, *FOXG1* (forkhead box G1) was selected with the highest absolute average weight. This gene is associated with colon cancer and can be one of the therapeutic targets [75]. *XKR6* (XK-related 6), which came second on the list, is associated with metastasis in colon cancer [76]. Another selected gene was *FSCN1* (fascin actin-bundling protein 1). The overexpression of this gene is associated with colon cancer. High tumor expression of *EGFL7* (epidermal growth factor-like domain 7) has been associated with poor prognosis in colon cancer [77], and this gene was also selected. These findings imply that sCCA is capable of selecting genes that are biologically relevant to the respective cancer type.

## 4. Discussion

The availability of numerous heterogeneous datasets on the same set of patients necessitates methods that go beyond visual consolidation of these data. Owing to the high dimensionality of omics data, the crux of the problem is to jointly reduce the dimension of datasets whilst preserving their complex correlation structure.

In this study, we employed sparse canonical correlation analysis to integrate TCGA gene expression and DNA methylation datasets. We performed clustering on the combined latent scores. Using a case study with three cancer datasets, we illustrated the performance in terms of survival analysis. All the identified subtypes were statistically significant (*p*-value ≤0.05). Hazard analysis indicated improved clinical association in comparison with both single- and multi-omics cancer molecular subtyping algorithms. Pathway analysis was performed to understand the pathway variation in each subtype, alongside the underlying biological mechanisms to elucidate potential drug targets. We also performed a brief analysis of the canonical vectors for gene expression datasets, which exhibited the ability of sCCA to select genes that are biologically pertinent to their respective cancer type.

In the future, this methodology could be extended to incorporate other types of genomics data into the analysis, such as microRNA (miRNA). A web-based server connected with TCGA could also be developed for use by the medical community. This server will allow users to input omics profiles for a patient and obtain their subtype classification, and this will require an additional layer of supervised learning.

## Figures and Tables

**Table 1:** Comparison with SNF and OTRIMLE in terms of log-rank *p*-value and minimum hazard ratio; sCCA demonstrates lower *p*-values and larger $HR_{min}$.

Data	Method	*p*-Value	Minimum Hazard Ratio	SEP
GBM	sCClust SNF OTRIMLE NEMO PINTMF	**0.00004** 0.00769 0.00462 0.00256 0.00646	**1.4907** 1.3509 1.1796 1.3513 1.1594	**1.2889** 1.2299 1.1496 1.2665 1.1880
LSCC	sCClust SNF OTRIMLE NEMO PINTMF	**0.00031** 0.01612 0.02126 0.00107 0.00444	**1.6106** 1.2356 1.1345 1.3859 1.4136	**1.7026** 1.4667 1.1756 1.5254 1.4793
COAD	sCClust SNF OTRIMLE NEMO PINTMF	**0.00041** 0.03923 0.03914 0.01260 0.00341	**8.7327** 4.8915 2.8059 5.7714 4.1594	**1041.07** 356.462 724.132 872.090 358.442

## Data Availability

The data used in this study are publicly available at the Cancer Genome Atlas (TCGA) website. Code to reproduce the analyses of the article is available at https://gitlab.com/muneeba.jilani/data-integration-project.

## Supplementary Materials

The following supporting information can be downloaded at: https://www.mdpi.com/article/10.3390/genes15050631/s1. Table S1: Datasets used in this study; Table S2: Sizes of the clusters; Table S3: Genes selected by sCCA; Figure S1: Elbow plots.

## Abbreviations

The following abbreviations are used in this manuscript:

TCGA	the Cancer Genome Atlas
GBM	glioblastoma multiforme
COAD	colon adenocarcinoma
LSCC	lung squamous cell carcinoma
sCCA	sparse canonical correlation analysis

## References

[1] Bird A. (2002). DNA methylation patterns and epigenetic memory. Genes Dev..

[2] Dhar G.A., Saha S., Mitra P., Nag Chaudhuri R. (2021). DNA methylation and regulation of gene expression: Guardian of our health. Nucleus.

[3] Moore L.D., Le T., Fan G. (2013). DNA methylation and its basic function. Neuropsychopharmacology.

[4] Xu W., Xu M., Wang L., Zhou W., Xiang R., Shi Y., Zhang Y., Piao Y. (2019). Integrative analysis of DNA methylation and gene expression identified cervical cancer-specific diagnostic biomarkers. Signal Transduct. Target. Ther..

[5] Wagner J.R., Busche S., Ge B., Kwan T., Pastinen T., Blanchette M. (2014). The relationship between DNA methylation, genetic and expression inter-individual variation in untransformed human fibroblasts. Genome Biol..

[6] Jiang L., Xiao Y., Ding Y., Tang J., Guo F. (2019). Discovering cancer subtypes via an accurate fusion strategy on multiple profile data. Front. Genet..

[7] Froeling F.E., Casolino R., Pea A., Biankin A.V., Chang D.K., Precision-Panc (2021). Molecular subtyping and precision medicine for pancreatic cancer. J. Clin. Med..

[8] Lin X., Tian T., Wei Z., Hakonarson H. (2022). Clustering of single-cell multi-omics data with a multimodal deep learning method. Nat. Commun..

[9] Rappoport N., Shamir R. (2019). NEMO: Cancer subtyping by integration of partial multi-omic data. Bioinformatics.

[10] Wu D., Wang D., Zhang M.Q., Gu J. (2015). Fast dimension reduction and integrative clustering of multi-omics data using low-rank approximation: Application to cancer molecular classification. BMC Genom..

[11] Nguyen T., Tagett R., Diaz D., Draghici S. (2017). A novel approach for data integration and disease subtyping. Genome Res..

[12] Yamada R., Okada D., Wang J., Basak T., Koyama S. (2021). Interpretation of omics data analyses. J. Hum. Genet..

[13] Zhang Z., Castelló A. (2017). Principal components analysis in clinical studies. Ann. Transl. Med..

[14] Soneson C., Lilljebjörn H., Fioretos T., Fontes M. (2010). Integrative analysis of gene expression and copy number alterations using canonical correlation analysis. BMC Bioinform..

[15] Zheng Y., Jun J., Brennan K., Gevaert O. (2023). Epimix is an integrative tool for epigenomic subtyping using dna methylation. Cell Rep. Methods.

[16] Arslanturk S., Draghici S., Nguyen T. (2019). Integrated cancer subtyping using heterogeneous genome-scale molecular datasets. Proceedings of the Pacific Symposium on Biocomputing 2020.

[17] ElKarami B., Alkhateeb A., Qattous H., Alshomali L., Shahrrava B. (2022). Multi-omics Data Integration Model Based on UMAP Embedding and Convolutional Neural Network. Cancer Inform..

[18] Qattous H., Azzeh M., Ibrahim R., Abed Al-Ghafer I., Al Sorkhy M., Alkhateeb A. (2023). PaCMAP-embedded convolutional neural network for multi-omics data integration. Heliyon.

[19] Csala A., Voorbraak F.P., Zwinderman A.H., Hof M.H. (2017). Sparse redundancy analysis of high-dimensional genetic and genomic data. Bioinformatics.

[20] R Core Team (2000). R Language Definition.

[21] Tajunisha S., Saravanan V. (2010). Performance analysis of k-means with different initialization methods for high dimensional data. Int. J. Artif. Intell. Appl. (IJAIA).

[22] Hotelling H. (1935). Canonical correlation analysis (CCA). J. Educ. Psychol..

[23] Witten D.M., Tibshirani R.J. (2009). Extensions of sparse canonical correlation analysis with applications to genomic data. Stat. Appl. Genet. Mol. Biol..

[24] Waaijenborg S., Zwinderman A.H. (2007). Penalized canonical correlation analysis to quantify the association between gene expression and DNA markers. Proceedings of the BMC Proceedings.

[25] Rodosthenous T., Shahrezaei V., Evangelou M. (2020). Integrating multi-OMICS data through sparse canonical correlation analysis for the prediction of complex traits: A comparison study. Bioinformatics.

[26] Tibshirani R. (1996). Regression shrinkage and selection via the Lasso. J. R. Stat. Soc. Ser. B (Methodol.).

[27] Lloyd S. (1982). Least squares quantization in PCM. IEEE Trans. Inf. Theory.

[28] Goel M.K., Khanna P., Kishore J. (2010). Understanding survival analysis: Kaplan-Meier estimate. Int. J. Ayurveda Res..

[29] Rich J.T., Neely J.G., Paniello R.C., Voelker C.C., Nussenbaum B., Wang E.W. (2010). A practical guide to understanding Kaplan-Meier curves. Otolaryngol.—Head Neck Surg..

[30] Rafique O., Mir A.H. (2020). A topological approach for cancer subtyping from gene expression data. J. Biomed. Inform..

[31] Blagoev K.B., Wilkerson J., Fojo T. (2012). Hazard ratios in cancer clinical trials—A primer. Nat. Rev. Clin. Oncol..

[32] Clark T.G., Bradburn M.J., Love S.B., Altman D.G. (2003). Survival analysis part I: Basic concepts and first analyses. Br. J. Cancer.

[33] Prentice R.L., Gloeckler L.A. (1978). Regression analysis of grouped survival data with application to breast cancer data. Biometrics.

[34] Ng’andu N.H. (1997). An empirical comparison of statistical tests for assessing the proportional hazards assumption of Cox’s model. Stat. Med..

[35] Grambsch P.M., Therneau T.M. (1994). Proportional hazards tests and diagnostics based on weighted residuals. Biometrika.

[36] In J., Lee D.K. (2019). Survival analysis: Part II-applied clinical data analysis. Korean J. Anesthesiol..

[37] Royston P., Sauerbrei W. (2004). A new measure of prognostic separation in survival data. Stat. Med..

[38] Ng A. (2012). Clustering with the k-means algorithm. Mach. Learn..

[39] Shahapure K.R., Nicholas C. Cluster quality analysis using silhouette score. Proceedings of the 2020 IEEE 7th International Conference on Data Science and Advanced Analytics (DSAA).

[40] Edwards A.W., Cavalli-Sforza L.L. (1965). A method for cluster analysis. Biometrics.

[41] Sidaway P. (2017). Glioblastoma subtypes revisited. Nat. Rev. Clin. Oncol..

[42] Liu J., Jiang C., Xu C., Wang D., Shen Y., Liu Y., Gu L. (2021). Identification and development of a novel invasion-related gene signature for prognosis prediction in colon adenocarcinoma. Cancer Cell Int..

[43] Polo V., Pasello G., Frega S., Favaretto A., Koussis H., Conte P., Bonanno L. (2016). Squamous cell carcinomas of the lung and of the head and neck: New insights on molecular characterization. Oncotarget.

[44] Emmert-Streib F., Dehmer M. (2019). Introduction to survival analysis in practice. Mach. Learn. Knowl. Extr..

[45] Wang B., Mezlini A.M., Demir F., Fiume M., Tu Z., Brudno M., Haibe-Kains B., Goldenberg A. (2014). Similarity network fusion for aggregating data types on a genomic scale. Nat. Methods.

[46] Pai S., Bader G.D. (2018). Patient similarity networks for precision medicine. J. Mol. Biol..

[47] Hershberg E.A., Stevens G., Diesh C., Xie P., De Jesus Martinez T., Buels R., Stein L., Holmes I. (2021). JBrowseR: An R interface to the JBrowse 2 genome browser. Bioinformatics.

[48] Ou J., Zhu L.J. (2019). trackViewer: A Bioconductor package for interactive and integrative visualization of multi-omics data. Nat. Methods.

[49] Xu T., Le T.D., Liu L., Su N., Wang R., Sun B., Colaprico A., Bontempi G., Li J. (2017). CancerSubtypes: An R/Bioconductor package for molecular cancer subtype identification, validation and visualization. Bioinformatics.

[50] Pierre-Jean M., Mauger F., Deleuze J.F., Le Floch E. (2022). PIntMF: Penalized Integrative Matrix Factorization method for multi-omics data. Bioinformatics.

[51] Zhang E., Zhang M., Shi C., Sun L., Shan L., Zhang H., Song Y. (2020). An overview of advances in multi-omics analysis in prostate cancer. Life Sci..

[52] Coretto P., Serra A., Tagliaferri R. (2018). Robust clustering of noisy high-dimensional gene expression data for patients subtyping. Bioinformatics.

[53] Ramanan V.K., Shen L., Moore J.H., Saykin A.J. (2012). Pathway analysis of genomic data: Concepts, methods, and prospects for future development. Trends Genet..

[54] Lee E., Chuang H.Y., Kim J.W., Ideker T., Lee D. (2008). Inferring pathway activity toward precise disease classification. PLoS Comput. Biol..

[55] Kanehisa M. (2002). The KEGG database. Proceedings of the Novartis Foundation Symposium.

[56] Oh J.H., Choi W., Ko E., Kang M., Tannenbaum A., Deasy J.O. (2021). PathCNN: Interpretable convolutional neural networks for survival prediction and pathway analysis applied to glioblastoma. Bioinformatics.

[57] Wang J.J., Wang H., Zhu B.L., Wang X., Qian Y.H., Xie L., Wang W.J., Zhu J., Chen X.Y., Wang J.M. (2021). Development of a prognostic model of glioma based on immune-related genes. Oncol. Lett..

[58] Li Z., Zheng Z., Ruan J., Li Z., Tzeng C.M. (2016). Chronic inflammation links cancer and Parkinson’s disease. Front. Aging Neurosci..

[59] Savaskan N.E., Fan Z., Broggini T., Buchfelder M., Eyupoglu I.Y. (2015). Neurodegeneration in the brain tumor microenvironment: Glutamate in the limelight. Curr. Neuropharmacol..

[60] Jin X., Guan Y., Sheng H., Liu Y. (2017). Crosstalk in competing endogenous RNA network reveals the complex molecular mechanism underlying lung cancer. Oncotarget.

[61] Zhan X., Lu M., Yang L., Yang J., Zheng S., Guo Y., Li B., Wen S., Li J., Li N. (2022). Ubiquitination-mediated molecular pathway alterations in human lung squamous cell carcinomas identified by quantitative ubiquitinomics. Front. Endocrinol..

[62] Tran M.T. (2021). Overview of Ca2+ signaling in lung cancer progression and metastatic lung cancer with bone metastasis. Explor. Target. Anti-Tumor Ther..

[63] Bodaghi S., Yamanegi K., Xiao S.Y., Da Costa M., Palefsky J.M., Zheng Z.M. (2005). Colorectal papillomavirus infection in patients with colorectal cancer. Clin. Cancer Res..

[64] Kolodkin-Gal D., Zamir G., Edden Y., Pikarsky E., Pikarsky A., Haim H., Haviv Y.S., Panet A. (2008). Herpes simplex virus type 1 preferentially targets human colon carcinoma: Role of extracellular matrix. J. Virol..

[65] Wen S., He L., Zhong Z., Mi H., Liu F. (2020). Prognostic model of colorectal cancer constructed by eight immune-related genes. Front. Mol. Biosci..

[66] Mjelle R., Sjursen W., Thommesen L., Sætrom P., Hofsli E. (2019). Small RNA expression from viruses, bacteria and human miRNAs in colon cancer tissue and its association with microsatellite instability and tumor location. BMC Cancer.

[67] Arunachalam E., Rogers W., Simpson G.R., Möller-Levet C., Bolton G., Ismael M., Smith C., Keegen K., Bagwan I., Brend T. (2022). HOX and PBX gene dysregulation as a therapeutic target in glioblastoma multiforme. BMC Cancer.

[68] Cimino P.J., Kim Y., Wu H.J., Alexander J., Wirsching H.G., Szulzewsky F., Pitter K., Ozawa T., Wang J., Vazquez J. (2018). Increased HOXA5 expression provides a selective advantage for gain of whole chromosome 7 in IDH wild-type glioblastoma. Genes Dev..

[69] Ferletta M., Uhrbom L., Olofsson T., Pontén F., Westermark B. (2007). Sox10 has a broad expression pattern in gliomas and enhances platelet-derived growth factor-B–induced gliomagenesis. Mol. Cancer Res..

[70] Chen B., Liang T., Yang P., Wang H., Liu Y., Yang F., You G. (2016). Classifying lower grade glioma cases according to whole genome gene expression. Oncotarget.

[71] Xie J., Qiao L., Deng G., Liang N., Xing L., Zhang J. (2022). PCGF1 is a prognostic biomarker and correlates with tumor immunity in gliomas. Ann. Transl. Med..

[72] Plowman J., Bolderson E., Burgess J., Richard D., O’Byrne K. (2019). P2. 14-08 Banf1 Predicts Lung Cancer Survival and Sensitivity to Platinum-Based Chemotherapy. J. Thorac. Oncol..

[73] Liu H.Y., Zhao H., Li W.X. (2019). Integrated analysis of transcriptome and prognosis data identifies FGF22 as a prognostic marker of lung adenocarcinoma. Technol. Cancer Res. Treat..

[74] Shin G.C., Moon S.U., Kang H.S., Choi H.S., Han H.D., Kim K.H. (2019). PRKCSH contributes to tumorigenesis by selective boosting of IRE1 signaling pathway. Nat. Commun..

[75] Wu H., Qian C., Liu C., Xiang J., Ye D., Zhang Z., Zhang X. (2018). Role and mechanism of FOXG1 in invasion and metastasis of colorectal cancer. Sheng Wu Gong Cheng Xue Bao Chin. J. Biotechnol..

[76] Shen P.C., Wang Y.F., Chang H.C., Huang W.Y., Lo C.H., Su Y.F., Yang J.F., Lin C.S., Dai Y.H. (2022). Developing a novel DNA methylation risk score for survival and identification of prognostic gene mutations in endometrial cancer: A study based on TCGA data. Jpn. J. Clin. Oncol..

[77] Hansen T.F., Andersen R.F., Olsen D.A., Sørensen F.B., Jakobsen A. (2017). Prognostic importance of circulating epidermal growth factor-like domain 7 in patients with metastatic colorectal cancer treated with chemotherapy and bevacizumab. Sci. Rep..

