# Supplementary material for: Elucidating Cancer Subtypes by Using the Relationship between DNA Methylation and Gene Expression
Source: Genes (Basel). 2024 May 16;15(5):631. doi: 10.3390/genes15050631 (PMC11121157; doi:10.3390/genes15050631)
Supplement: Supplementary file 1 [file genes-15-00631-s001.zip › genes-2994072-supplementary.pdf]

Elucidating Subtypes by using the Relationship between DNA Methylation and Gene Expression in Cancer – Supplementary Material

Table S1: Datasets used in this study: Gene expression and DNA methylation datasets corresponding to Glioblastoma Multiforme (GBM), lung squamous cell carcinoma (LSCC) and colon adenocarcinoma (COAD) cancers

Data	Samples	Expression Dimensionality	Methylation Dimensionality
GBM	211	12042	13050
LSCC	106	12042	23074
COAD	92	17814	23088

Table S2: Sizes of clusters for Glioblastoma Multiforme (GBM), lung squamous cell carcinoma (LSCC) and colon adenocarcinoma (COAD) cancers

Data	Clusters
GBM	150, 44, 17
LSCC	38, 32, 24, 12
COAD	45, 26, 21

Table S3: Genes selected by sCCA for Glioblastoma Multiforme (GBM), lung squamous cell carcinoma (LSCC) and colon adenocarcinoma (COAD) cancers

Data	Genes
GBM	*HOXA6, HOXA5, SOX10, TEX11, TLR5, HOXA7, SERTAD3, TIMP1, DYNLT3, ST7L, HOXD3, HOXA4, CASP8, LGALS3, FLJ13611, EMP3, NME6, CBLN1, HOXC10, HRASLS, MYO1F, CSF1R, GAL3ST1, TUBB2A, IL10RA, DNAJC17, DRD4, KCNS3, C1orf165, WAS*
LSCC	*PCGF1, BANF1, FGF22, BAT1, GPR32, PRKCSH, SBNO2, FKBP8, HIRA, NACAP1, GTF2H4, KLHL1, LOC90379, E2F4, PFDN6, SCYL2, WDR57, ATN1, FLJ21075, CD3E, CASP2, GNL3L, AIP, C20orf10, OTOF, ZNF747, TRIP10, TBL1XR1, ADAM30, PPP3R1, AQP7, PPIL2, MYBBP1A, EMD, RRP12, SEMA6A, TM4SF20, GMEB1, ACTR8, DLST*
COAD	*FOXG1, XKR6, UBE2G1, ZNF141, EPM2AIP1, FSCN1, RAB5C, EGFL7, MGC42090, TRIM7, NXF3, SEMG1, MRFAP1L1, RAB32, C9orf58, PLP1, C7orf47, MTERF, LCE2C, RSPO3, WDR82, GPR143, NUTF2, CNO, GPR42, ZNF256, P2RY14, GJA3, TACR3, CPEB3, SERPINB10, C18orf8, NOTCH3, ANXA10, RNF19B, QPRT, VPRBP, PATE, EIF5A, CH25H, CLASP1, C10orf47, PPP6C, SALL3, RFX4, NUBP1, HOMER2, OR1L4, ANKRD5, PRPSAP2, SPEG, DNALI1, GPR126, FAM69B, ZIC5, AMFR, FBF1, SPATS2, C6orf97, TUBG1, NDNL2, MLH1, CX3CL1, HOXB8, ZNF304, N4BP1, DCTN2, IL9, KHDRBS3, SH3GL1, OR8B12, GPR34*

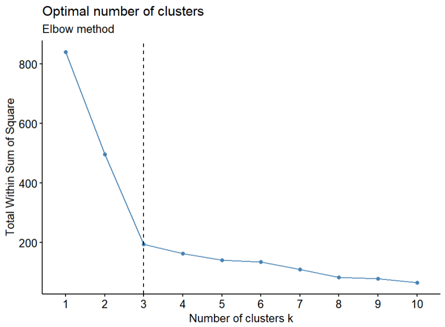

(a) GBM Cancer

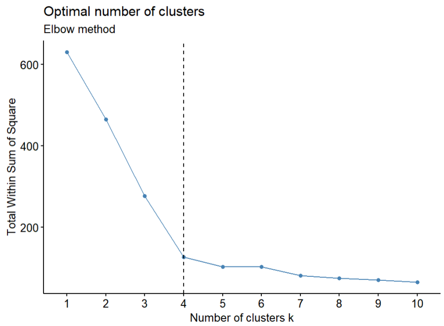

(b) Lung Cancer

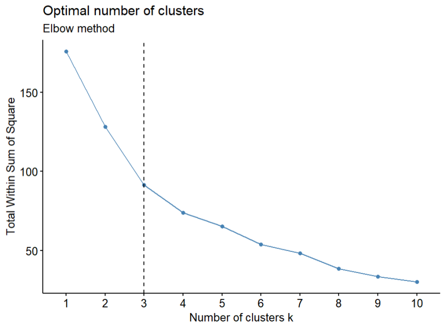

(c) Colon Cancer

Figure S1: Elbow plots for for (a) Glioblastoma Multiforme (GBM), (b) lung squamous cell carcinoma (LSCC) and (c) colon adenocarcinoma (COAD) can-cers
